# Potassium Dependent Regulation of Astrocyte Water Permeability Is Mediated by cAMP Signaling

**DOI:** 10.1371/journal.pone.0034936

**Published:** 2012-04-06

**Authors:** Yutong Song, Eli Gunnarson

**Affiliations:** Department of Women’s and Children’s Health, Karolinska Institutet, Astrid Lindgren Children’s Hospital, Stockholm, Sweden; Dalhousie University, Canada

## Abstract

Astrocytes express potassium and water channels to support dynamic regulation of potassium homeostasis. Potassium kinetics can be modulated by aquaporin-4 (AQP4), the essential water channel for astrocyte water permeability regulation. We investigated whether extracellular potassium ([K^+^]_o_) can regulate astrocyte water permeability and the mechanisms of such an effect. Studies were performed on rat primary astrocytes and a rat astrocyte cell line transfected with AQP4. We found that 10mM [K^+^]_o_ caused an immediate, more than 40%, increase in astrocyte water permeability which was sustained in 5min. The water channel AQP4 was a target for this regulation. Potassium induced a significant increase in intracellular cAMP as measured with a FRET based method and with enzyme immunoassay. We found that protein kinase A (PKA) could phosphorylate AQP4 *in vitro*. Further elevation of [K^+^]_o_ to 35mM induced a global intracellular calcium response and a transient water permeability increase that was abolished in 5min. When inwardly rectifying potassium (Kir)-channels were blocked, 10mM [K^+^]_o_ also induced a calcium increase and the water permeability increase no longer persisted. In conclusion, we find that elevation of extracellular potassium regulates AQP4 and astrocyte water permeability via intracellular signaling involving cAMP. A prolonged increase of astrocyte water permeability is Kir-channel dependent and this response can be impeded by intracellular calcium signaling. Our results support the concept of coupling between AQP4 and potassium handling in astrocytes.

## Introduction

Extracellular potassium in the brain has to be tightly controlled. Astrocytes are essential for brain potassium homeostasis and are responsible for regulating potassium dynamics following neuronal synaptic activity [1], [2]. During intensive neuronal firing, the extracellular potassium concentration ([K^+^]_o_) is estimated to increase from the basal level of about 2.5mM up to 8mM, with a ceiling level of 12mM [3], [4], followed by a rapid [K^+^]_o_ restoration governed by glial cells [5], [6]. When the systems for maintaining potassium homeostasis are disrupted or overwhelmed, extracellular potassium concentrations can reach values as high as 30 to 80mM [7]. This can be predicted to occur in pathological conditions and leads to severely compromised CNS function [8], [9].

Astrocytes express the water channel AQP4 that is suggested to be intimately linked to potassium homeostasis, as rapid water transport via the channel is expected to facilitate ion fluxes. A functional link between water transport via AQP4 and potassium has been supported by the finding of slowed potassium kinetics *in vivo* in mice lacking AQP4 [10], [11]. As the conductance of inwardly rectifying K-channels (Kir-channels) is known to be essential for astrocyte potassium permeability [12], a functional relationship between Kir-channels and aquaporins in astrocytes has been debated [13], [14], [15]. We previously reported that APQ4 water permeability in astrocytes can be dynamically regulated [16]. However, it has not been demonstrated whether astrocyte AQP4 responds to changes in extracellular potassium. Here, we raised the question whether changes in extracellular potassium will induce dynamic regulation of AQP4 and astrocyte water permeability. We show that elevations in potassium increase astrocyte water permeability via cAMP-dependent mechanism involving AQP4. A prolonged upregulation of astrocyte water permeability is dependent on Kir-channel function. The effect can be modulated by calcium when such signaling is triggered by extracellular potassium.

**Figure 1:**
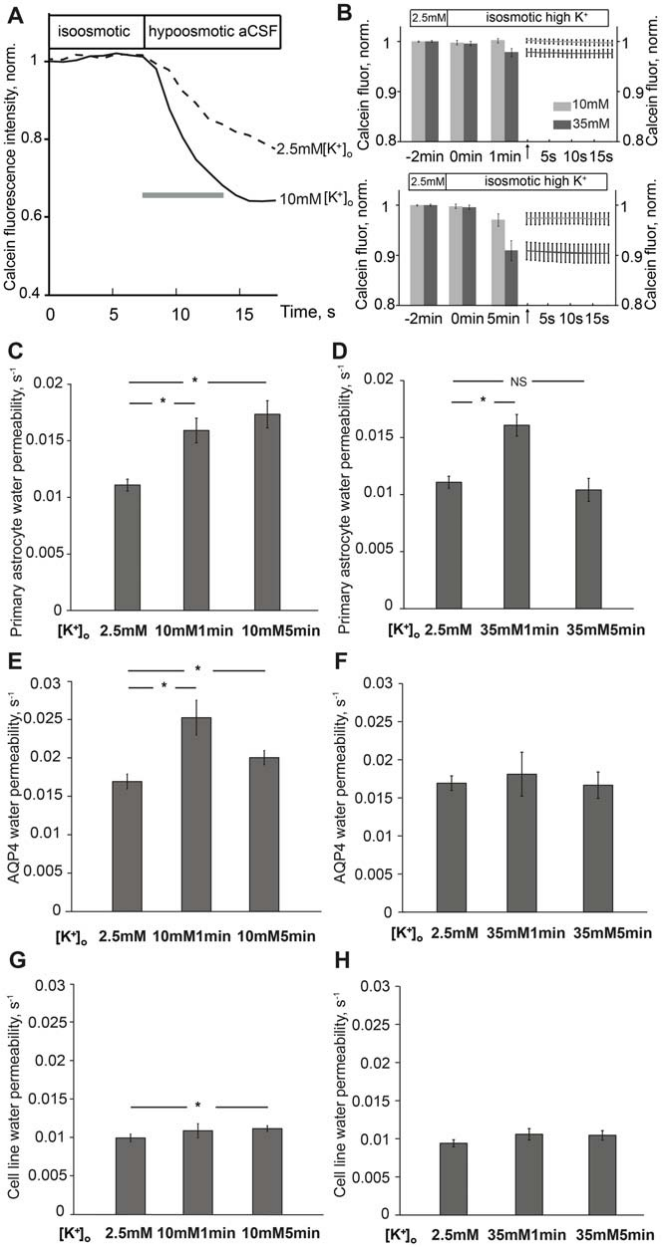
Elevated potassium regulates astrocyte water permeability. (A) Representative recordings from two independent primary astrocytes showing changes in calcein fluorescence (2.5mM potassium, dashed line; 10mM potassium, solid line) in water permeability measurements. The grey bar depicts the part of the curve used for calculation of plasma membrane water permeability (s; seconds). (B) Fluorescence intensity change induced by isosmotic high potassium: bars show quantified fluorescence intensity at 2min before high potassium (-2min), at start (0min) and after 1min and 5min of indicated potassium concentrations, respectively. Values were normalized to the initial intensity at 2.5mM potassium. Right hand panels show fluorescence intensity curves for isosmotic high potassium (grey: 10mM; black: 35mM) within the time window (∼20s) of water permeability recordings after 1 or 5 minutes. Arrows indicate starting points. Values are means and error bars of each individual acquired time point, n = 48–65. (C) Water permeability in primary astrocytes exposed to 10mM potassium for 1min and 5min, respectively. 10mM potassium caused a significant water permeability increase at 1min and 5min (*p<0.001), n = 57–92. (D) 35mM potassium caused a transient astrocyte water permeability increase at 1min (*p<0.001). The effect was abolished after 5min (NS; not significant p = 0.52), n = 53–92. (E) AQP4 specific water permeability calculated from astrocyte cell line. 10mM potassium significantly increased AQP4 water permeability at 1min (*p<0.001) and 5min (*p<0.05), N = 5-7. (F) 35mM potassium did not increase AQP4 water permeability either at 1min (p = 0.62) or 5min (p = 0.89), N = 5–16. (G) Water permeability in AQP4-negative astrocyte cell line. 10mM potassium for 1min did not have any effect on water permeability in AQP4-negative cells. A small but significant water permeability was observed after 5min of 10mM potassium (P = 0.045), n = 43–130. (H) 35mM potassium did not have any effect on water permeability in AQP4-negative astrocyte cell line (1min, p = 0.62; 5min p = 0.89), n = 33–119. Values are means±SEM, n; number of cells, N; number of plates.

## Results

### Elevated Potassium Regulates Astrocyte Water Permeability

To explore the effect of elevated [K^+^]_o_ on plasma membrane water permeability in astrocytes, we applied two extracellular potassium concentration elevations; from 2.5mM to 10mM or 35mM [K^+^]_o_. Representative recordings from primary astrocytes exposed to 2.5mM and 10mM [K^+^]_o_, respectively, are shown in Figure 1A. Control experiments showed that changes in fluorescence induced by isosmotic high potassium did not affect water permeability measurements (Figure 1B and Materials and Methods). In primary astrocytes, 10mM [K^+^]_o_ for 1min caused a 46% increase in water permeability (0.011 ± 0.0005 versus 0.016 ± 0.0011, p<0.001). Following exposure to 10mM [K^+^]_o_ for 5min, astrocyte water permeability was increased by 55% (0.011 ± 0.0005 versus 0.017 ± 0.0012, p<0.001) (Figure 1C). As for 10mM [K^+^]_o_, exposure to 35mM [K^+^]_o_ for 1min caused a 46% increase in water permeability (0.011 ± 0.0005 versus 0.016 ± 0.0009, p<0.001) in primary astrocytes. Conversely, when astrocytes were exposed to 35mM [K^+^]_o_ for 5min, astrocyte water permeability was no longer increased (0.011 ± 0.0005 versus 0.010 ± 0.0010, p = 0.52) (Figure 1D).

To explore whether the potassium-dependent upregulation of astrocyte water permeability is attributed to an effect on AQP4, we analyzed the effect of potassium on AQP4-positive and AQP4-negative cells in the astrocyte cell line. This enables us to determine the specific AQP4 water channel permeability. In the transfected astrocyte cell line, 10mM [K^+^]_o_ for 1min caused a 56% increase in AQP4 water permeability (0.016 ± 0.0009 versus 0.025 ± 0.0022, p<0.001). After 5min of 10mM [K^+^]_o_, AQP4 water permeability was increased by 25% (0.016 ± 0.0009 versus 0.020 ± 0.0009, p<0.05) (Figure 1E). Exposure to 35mM [K^+^]_o_ for 1 or 5min did not cause any significant increase in AQP4 water permeability (0.016 ± 0.0009 versus 0.018 ± 0.0029, p = 0.62 and 0.016 ± 0.0009 versus 0.017 ± 0.0017, p = 0.89, respectively) (Figure 1F). In cells that did not express AQP4 (AQP4-negative cells), there was no effect on water permeability of either 10mM [K^+^]_o_ or 35mM [K^+^]_o_ (10mM 1min, 0.009 ± 0.0005 versus 0.010 ± 0.0009, p = 0.34; 35mM 1min, 0.009 ± 0.0005 versus 0.011 ± 0.0008 p = 0.21; 35mM 5min, 0.009 ± 0.0005 versus 0.010 ± 0.0006 p = 0.24)( Figures 1G, 1H), except in AQP4-negative cells exposed to 10mM [K^+^]_o_ for 5min where there was a slight but significant increase in water permeability (0.009 ± 0.0005 versus 0.011 ± 0.0003, p = 0.045) (Figure 1G). The data support that the observed immediate potassium-induced increase in astrocyte water permeability is mediated by AQP4.

### High Potassium Induces cAMP Production and Increases Astrocyte Water Permeability via PKA-dependent Regulation

**Figure 2:**
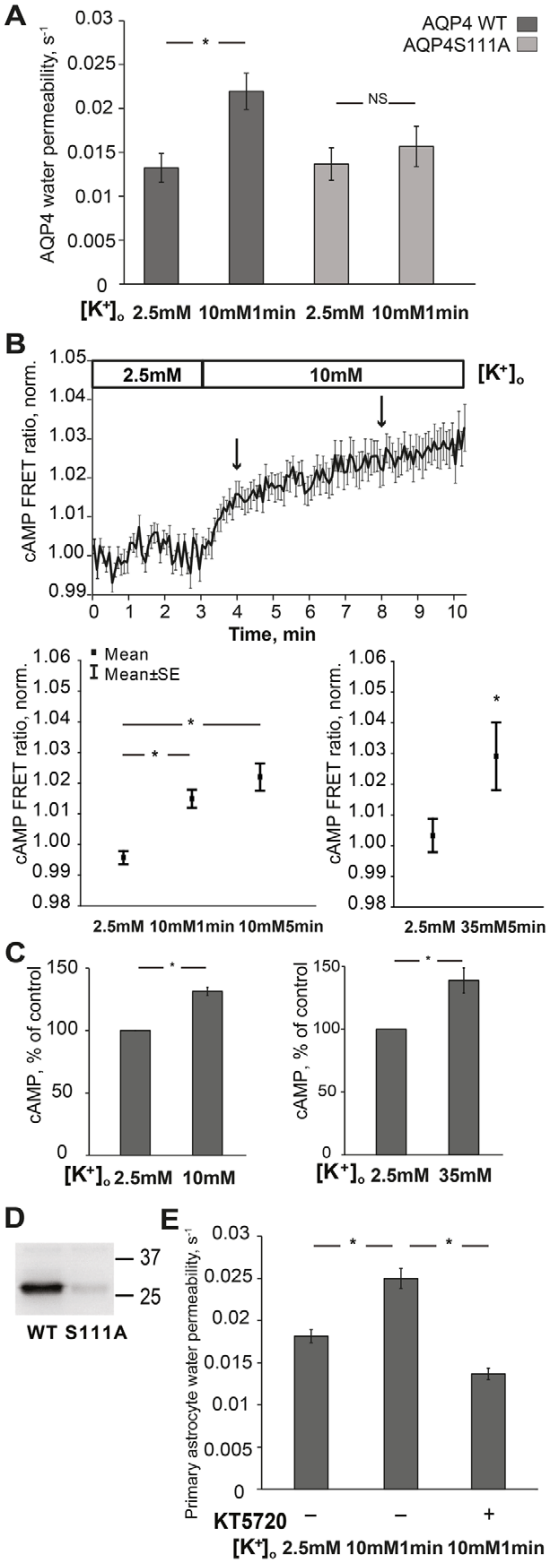
Potassium elevation induces cAMP production and increases astrocyte water permeability via PKA-dependent phosphorylation of AQP4. (A) 10mM potassium significantly increased WT AQP4 water permeability (*p<0.01), N = 6, but did not increase S111A AQP4 water permeability (NS; not significant p = 0.51), N = 6. Dark grey bars: WT AQP4, light grey bars: S111A AQP4. (B) Upper panel shows plot of mean values of normalized FRET ratio (donor/acceptor) with SE bars at every acquired point. Lower panel (left): cAMP FRET ratio at different time points: 2.5mM (at 3min in upper panel); 10mM 1min (arrow at 4min in upper panel); 10mM 5min (arrow at 8min in upper panel). 10mM potassium significantly increased cAMP FRET ratio in astrocyte cell line (*p<0.001), n = 26. Lower panel (right): 35mM potassium increased cAMP FRET ratio (*p<0.01), n =  16. (C) By using cAMP EIA kit, 10mM potassium significantly increased cAMP levels in primary cultured astrocytes (left) (*p<0.001), N = 7. 35mM potassium also increased cAMP levels in primary cultured astrocytes (right) (*p<0.001), N = 5. (D) Activated PKA phosphorylated the WT AQP4 peptide containing serine 111 *in vitro*, but failed to phosphorylate the S111A AQP4 peptide where serine 111 was substituted to alanine. Numbers indicate positions of molecular weight markers. (E) Incubation with the PKA inhibitor KT5720 abolished water permeability increase caused by 10mM potassium (*p<0.001), n = 126–152. Values are means±SEM, n; number of cells, N; number of plates.

We explored the role of the AQP4 serine 111 residue in the upregulation of AQP4 water permeability induced by high [K^+^]_o_. The astrocyte cell line was transfected with either wild type AQP4 (WT AQP4) or AQP4 S111A, where serine 111 had been substituted to alanine. The water permeability of WT AQP4 and AQP4 S111A was similar in 2.5mM potassium control aCSF (0.013 ± 0.0016 versus 0.013 ± 0.0018) (Figure 2A). As opposed to the increased water permeability observed in cells expressing WT AQP4, 10mM [K^+^]_o_ for 1min did not induce any significant change in mutant AQP4 S111A water permeability (0.013 ± 0.0018 versus 0.015 ± 0.0023, p = 0.41) (Figure 2A). This finding indicates that the residue serine 111 is a target for the potassium dependent regulation of AQP4 water permeability.

Next we tested the possibility that high extracellular potassium can induce cAMP production in astrocytes. As a second messenger cAMP has been implicated in astrocyte signaling in the brain when exposed to high [K^+^]_o_ [17], [18]. When cells were exposed to 10mM [K^+^]_o_, cAMP FRET ratio showed a significant change, indicating increased cAMP production, at 1min (1.9% ± 0.4%, p<0.001) and at 5min (2.5% ± 0.4%, p<0.001) in the astrocyte cell line (Figure 2B). Although we found no effect of 5min exposure to 35mM [K^+^]_o_ on AQP4 water permeability, this concentration did induce an increase in cAMP FRET ratio in the astrocyte cell line (2.5% ± 0.8%, p<0.01) (Figure 2B).

As primary cultured astrocytes had a low transfection rate using the FRET method, we used a cAMP EIA kit to assess the effect of potassium on cAMP in these cells. As opposed to dynamic, real-time responses measured by the FRET-based sensor method, this method records cAMP production directly *in vitro*. Application of 10mM [K^+^]_o_ and 35mM [K^+^]_o_ for 5min increased cAMP levels by 31% (31.4% ± 3.3%, p<0.001) and 43% (42.8% ± 9.5% in 37^o^C, 5min p<0.001), respectively (Figure 2C). As cAMP is the prime activator of protein kinase A (PKA) we tested whether PKA could phosphorylate AQP4. Two recombinant peptides, corresponding to residues 96–123 of WT AQP4 and of the AQP4 S111A mutant, respectively, were used for *in vitro* phosphorylation. Activated PKA phosphorylated the WT AQP4 peptide, but not the AQP4 S111A peptide, indicating that serine 111 is a potential site of PKA phosphorylation (Figure 2D).

Next we applied the selective PKA inhibitor KT5720 and measured the effect of 10mM [K^+^]_o_ on water permeability in primary astrocytes. Again, 10mM [K^+^]_o_ caused a 40% increase in water permeability (0.018 ± 0.0008 versus 0.025 ± 0.0012, p<0.001). Preincubation with KT5720 for 5min completely abolished the increase in astrocyte water permeability caused by 10mM [K^+^]_o_ (0.025 ± 0.0012 versus 0.014 ± 0.0007, p<0.001) (Figure 2E).

### Highly Elevated Potassium Prevents Astrocyte Water Permeability Increase via Intracellular Calcium Signaling

**Figure 3:**
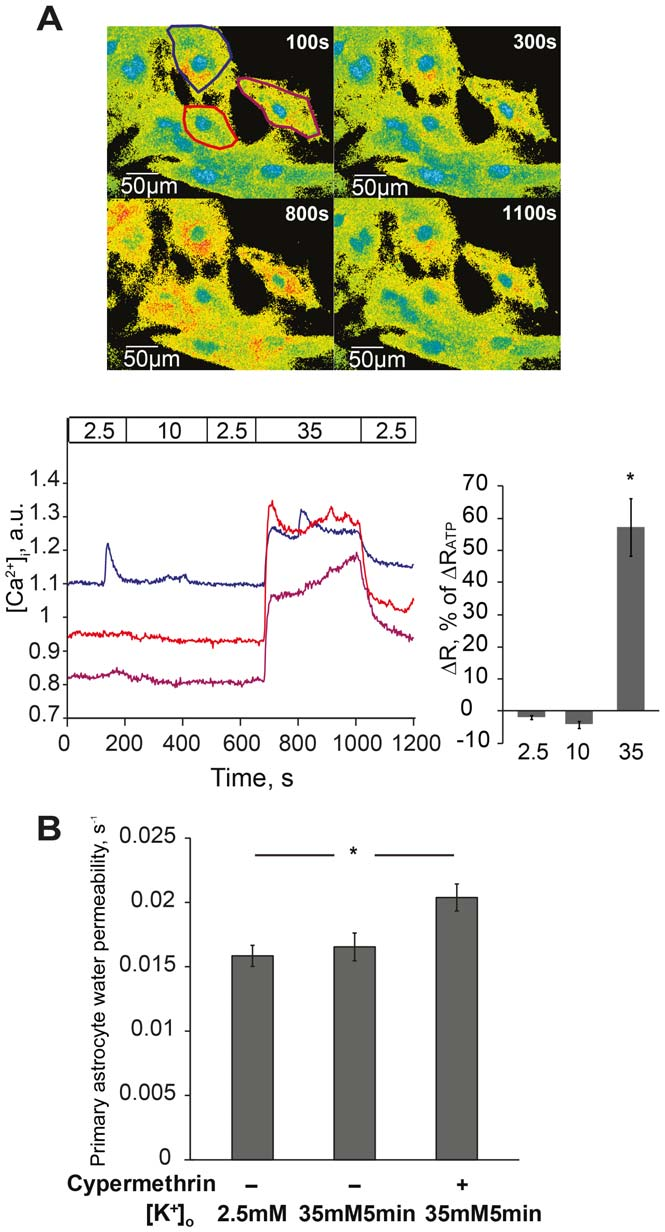
Highly elevated potassium prevents astrocyte water permeability increase via calcium signaling. (A) Upper panel shows representative 340/380nm fluorescence ratio images of astrocyte primary cultures loaded with Fura-2 AM at indicated time points, respectively. Lower left panel shows representative curves of intracellular calcium ([Ca^2+^]_i_) recordings in three individual cells during perfusion with potassium (marked with matched colors in upper panel). Potassium concentrations (mM) are marked in the horizontal bar. Arbitrary units (a.u.) represent ratio values corresponding to [Ca^2+^]_i_ changes. 10mM potassium (∼200–500s) did not cause any change in [Ca^2+^]_i_. 35mM potassium (∼680–1000s) induced a global [Ca^2+^]_i_ increase. The [Ca^2+^]_i_ increase disappeared when potassium was returned to baseline (2.5mM). One cell (blue) exhibits a spontaneous [Ca^2+^]_i_ peak at about 180s. Lower right panel shows summarized calcium data normalized to the peak [Ca^2+^]_i_ induced by ATP, n = 103. (B) After preincubation with the calcineurin inhibitor cypermethrin, 35mM potassium significantly increased astrocyte water permeability at 5min (*p<0.01, n = 73–89). Values are means±SEM, n; number of cells.

The profile of cAMP production did not differ between 10 and 35mM potassium and thus could not explain the different findings in water permeability. Interestingly, intracellular calcium signaling has been shown to be triggered by potassium with a concentration threshold of about 20–25mM, i.e. between the concentrations used in this study [19]. We recorded intracellular calcium to further explore the mechanisms for the different water permeability responses. In primary astrocytes loaded with Fura-2AM, no calcium response was observed following exposure to 10mM [K^+^]_o_ (Figure 3A). In contrast, exposure to 35mM [K^+^]_o_ caused an immediate global intracellular calcium increase in a vast majority of cells (Figure 3A). Quantification of the data showed that the calcium responses were all-or-none rather than dose-dependent (Figure 3A), consistent with other reports [19]. The change in intracellular calcium caused by 35mM [K^+^]_o_ was 57% (57.2+8.9%, p<0.001) relative to the calcium response to ATP (Figure 3A). The finding of a calcium response led us to hypothesize that the absence of increased astrocyte water permeability following 35mM [K^+^]_o_ could be due to a calcium-dependent dephosphorylation of AQP4. The protein phosphatase 2B, calcineurin, is activated by calcium [20]. Indeed, following 15min preincubation with cypermethrin (5nM), a potent inhibitor of calcineurin, astrocyte water permeability recorded after 5min of 35mM [K^+^]_o_ was increased by 25% (0.016 ± 0.0008 versus 0.020 ± 0.0010, p<0.05) (Figure 3B).

### Functional Relationship between Kir-channels and Potassium Effect on Astrocyte Water Permeability

**Figure 4:**
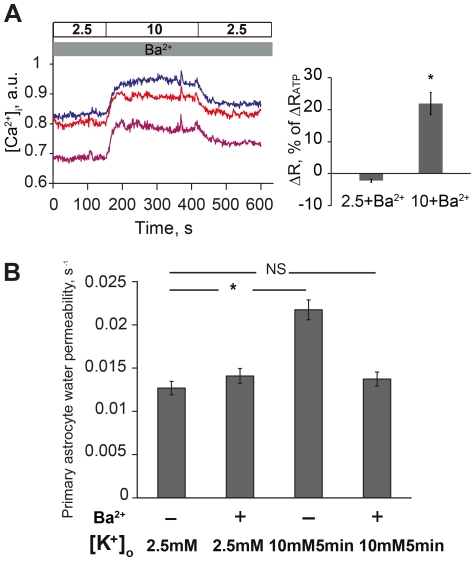
Functional relationship between Kir-channels and potassium effect on astrocyte water permeability. (A) Left panel shows representative recordings of intracellular calcium ([Ca^2+^]_i_) in three individual primary astrocytes loaded with Fura-2 AM during perfusion with indicated concentrations of potassium (mM) and 100µM barium (Ba^2+^). With barium, 10mM potassium triggered a global [Ca^2+^]_i_ increase (∼150–420s). Right panel shows summarized calcium data normalized to the peak [Ca^2+^]_i_ induced by ATP, n = 147. (B) The astrocyte water permeability increase caused by 10mM potassium was abolished when cells were preincubated with 100µM barium (*p<0.001). There was no difference in water permeability in control cells exposed to basal potassium concentrations with or without preincubation with barium (2.5mM potassium -/+ 100µM barium, p = 0.24), n = 60–89.

Kir-channel function is crucial for astrocyte potassium permeability, which in turn is important for voltage-dependent calcium responses [21]. Thus, we evaluated the role of Kir-channel function in the astrocyte calcium response induced by potassium. By applying 100µM barium, a concentration known to selectively block Kir-channels, a global intracellular calcium increase was obtained by 10mM [K^+^]_o_ in primary astrocytes (Figure 4A, see Figure 3A for comparison). The intracellular calcium change was 22% (21.9+3.5%, p<0.001) relative to the response to ATP (Figure 4A). As 10mM [K^+^]_o_ could, when Kir-channels were inhibited, induce a similar calcium response to the one observed with 35mM [K^+^]_o_, we investigated the effect of Kir-channel inhibition on astrocyte water permeability. After preincubation with barium, 10mM [K^+^]_o_ for 5min no longer caused an increase in astrocyte water permeability (0.013 ± 0.0008 versus 0.014 ± 0.0010; p = 0.35) (Figure 4B), supporting that the calcium response initiates a negative signaling pathway that will abolish astrocyte water permeability increase.

## Discussion

Astrocyte responses to neuronal activity have been widely studied, and a role of astrocytes in modulation of synaptic activity has emerged. A basic task of astrocytes is the dynamic regulation of potassium following neuronal activity. It is generally accepted that membrane water transport is crucial to accompany ion fluxes to maintain homeostasis [22]. The aim of this study was to explore the effect of potassium on astrocyte water permeability and to study the mechanisms involved. We report that extracellular potassium regulates astrocyte water permeability in a concentration and time-dependent manner that involves cAMP and that can be modified by Kir-channels via intracellular calcium.

Within the expected physiological range of extracellular potassium elevation, 10mM [K^+^]_o_ caused a significant increase in astrocyte water permeability. The increase was sustained after 5 minutes. The water permeability increase caused by high [K^+^]_o_ occurred via increased cAMP production in astrocytes, shown by real-time recordings of cAMP in living cells and *in vitro* measurements of cAMP in primary astrocytes. The water channel AQP4 mediated the effect on water permeability, as 10mM [K^+^]_o_ could cause a significant increase in specific AQP4 water permeability without affecting water permeability in AQP4-negative cells. We previously demonstrated that the residue serine 111 is the molecular target for dynamic, short term regulation of AQP4 water permeability [16]. The role of serine 111 was confirmed in this study by water permeability measurements with the mutant AQP4 S111A as well as *in vitro* phosphorylation experiments on AQP4 S111. In support of downstream activation of PKA, selective inhibition of PKA abolished the potassium effect on astrocyte water permeability. Following prolonged exposure to potassium there was a slight increase in water permeability in AQP4-negative cells, suggesting the involvement of other factors affecting astrocyte water permeability over time. Although dynamic regulation of AQP4 may be mediated by several mechanisms, the evidence presented here supports the concept of AQP4 regulation by phosphorylation. However, we cannot exclude that phosphorylation of other residues on AQP4 or other proteins also are involved in this process.

The findings indicate that local increases in extracellular potassium, occurring as a result of synaptic activity, impose an immediate upregulation of astrocyte water permeability mediated by AQP4. The increased astrocyte water permeability, allowing for bidirectional water flux via AQP4, can thus be predicted to have a role in dynamic regulation of potassium and promote fast restoration of the extracellular milieu for neuronal transmission. Mislocalization or knockout of AQP4 has, accordingly, been shown to slow down potassium kinetics in transgenic animals [10], [11], [23]. Further, the fast and dynamic volume changes occurring in activity-dependent astrocyte swelling [24] may require immediate regulation of astrocyte water permeability.

Extracellular levels of potassium can be expected to reach 30–80mM under certain pathological conditions [7]. Potassium concentrations in the range of 30–40 mM will induce cortical spreading depression [25], [26]. We explored the effect of 35mM [K^+^]_o_ on astrocyte water permeability. As opposed to the 10mM [K^+^]_o_, the increase in water permeability was not sustained after 5 minutes of 35mM [K^+^]_o_, in spite of increased cAMP levels caused by this concentration. These findings suggested that highly elevated potassium will activate negative signaling pathway(s) acting on astrocyte water permeability via regulation of AQP4. We did not find that 35mM [K^+^]_o_ increased AQP4 water permeability in the cell line model at any of the two time points. The different 1min responses in primary astrocytes and cell line, respectively, could be due to a faster negative response to high [K^+^]_o_ in the cell line; however other explanations cannot be ruled out. To investigate mechanisms involved in the negative regulation of water permeability by high [K^+^]_o_, we explored the intracellular calcium response. Several studies have investigated calcium responses to high [K^+^]_o_ in astrocytes [27], [28], [29], and both voltage-dependent calcium channels and the Na^+^/Ca^2+^ exchanger have been implicated in potassium-stimulated calcium influx [29], [30]. Here we found that 35mM [K^+^]_o_ induced a distinct intracellular calcium increase, which was absent following exposure to the lower, yet elevated, potassium concentration of 10mM. Interestingly, in neurons the calcium-dependent protein phosphatase calcineurin has been found to be associated with PKA via a common anchor protein [31]. Also, dephosphorylation by calcineurin has previously been shown to occur in astrocytes [20]. We found that inhibition of calcineurin allowed for 35mM [K^+^]_o_ to increase water permeability, which supports that calcium-activated dephosphorylation via calcineurin is involved in the negative signaling pathway. A recent study demonstrated that spreading depression following exposure to 40mM [K^+^]_o_ in neocortical slices mainly resulted in fast neuronal, rather than astrocyte, swelling [26]. This observation may thus partly be due to the inhibited water permeability increase in astrocytes, as shown here, at potassium concentrations in this range. We speculate that the lack of water permeability increase following sustained pathologically high [K^+^]_o_ will restrain astrocyte function with regard to ion homeostasis, slow down potassium dynamics and contribute to a prolonged increase in [K^+^]_o_.

In spite of the reported co-enrichment of AQP4 with Kir4.1 [15], [32], there has been controversy regarding a functional relationship between AQP4 and Kir-channels in the brain [13], [14]. Kir-channels are essential for the high potassium permeability in astrocytes [12]. Inhibition of Kir-currents leads to a remarkable depolarization in astrocytes [33], [34], [35] and, conversely, astrocyte hyperpolarization has been shown to be caused by PKA activation of Kir-channels [36]. Hence, as suggested by the present results, it is plausible that during elevations in extracellular potassium, voltage-dependent responses can be counteracted by functioning Kir-channels. Indeed, the high astrocyte potassium permeability has been proposed to mask voltage-dependent calcium influx [21], with up to 20–25mM [K^+^]_o_ appearing to represent a threshold for calcium responses [19]. Consistent with this we found that only 35mM [K^+^]_o_ could induce calcium signaling. However, when Kir-channels were inhibited 10mM [K^+^]_o_ was also able to trigger a similar calcium response and, consequently, astrocyte water permeability was no longer increased. Thus, our data support a functional interaction between Kir-channels and the regulation of astrocyte water permeability.

In a previous report functional interactions between AQP4 and Kir4.1 was investigated, but not supported [14]. Glial water permeability remained unchanged when inhibition or RNAi knockdown of Kir4.1 was performed [13]. We found a similar result under control conditions using aCSF containing 2.5mM [K^+^]_o_, where astrocyte AQP4 and Kir-channels appeared to be functionally isolated with regard to water permeability. However, when astrocytes were exposed to elevated [K^+^]_o_ a functional interaction between APQ4 and Kir-channels appeared. It should be noted that in the present study we did not explore which specific Kir-channel(s) that are responsible for the functional interaction with water permeability regulation. Besides Kir4.1, other Kir-channels have been reported to be expressed in astrocytes [37], [38].

**Figure 5:**
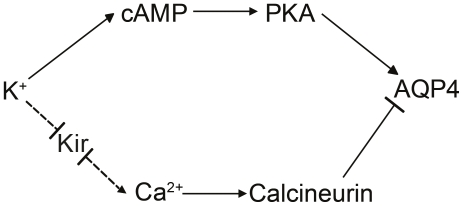
Potassium dependent regulation of astrocyte water permeability. Schematic illustration of the proposed signaling pathways involved in potassium regulation of astrocyte water permeability via AQP4.

In Figure 5 we present the proposed signaling pathways involved in the regulation of astrocyte water permeability via AQP4. High [K^+^]_o_ increases astrocyte water permeability via cAMP-dependent signaling. The water permeability response appears to depend on Kir-channels. When extracellular [K^+^]_o_ is further increased, a calcium-dependent regulation of AQP4 is activated.

In conclusion, we show evidence that astrocyte water permeability can respond to changes in extracellular potassium. The findings point to a functional coupling between water transport and potassium handling in astrocytes.

## Materials and Methods

### Ethics Statement

All experiments were approved by the Stockholm Northern Regional Ethical Committee, Stockholm, Sweden, and carried out in accordance to the Swedish National Law on ethical use of animals. Efforts were made to minimize the number of animals used and their suffering.

### Cell Cultures

Rat astrocyte cell line (DI TNC1), (European Collection of Cell Cultures, Centre for Applied Microbiology & Research, Salisbury, Wiltshire, UK) was grown on coverslips (Bioptechs, Butler, PA) in Dulbecco’s Modified Eagle’s medium (DMEM, Sigma Aldrich, Sweden) containing 50 U/mL penicillin and 50 µg/mL streptomycin supplemented with 10% fetal bovine serum (FBS), 0.11 mg/mL sodium pyruvate, and 4mM L-glutamine. The cells were transfected on the second day of culture with cDNA constructs encoding mouse AQP4 M23 (or mutant AQP4) tagged with green fluorescent protein (GFP) on the NH2-terminus using Exgene 500 reagent (Fermentas). The experiments were performed on the fourth day of culture, when the cells were subconfluent.

To obtain astrocytes in primary culture, rat cerebellum was dissected from Sprague-Dawley rat pups (B&K Universal, Sollentuna, Sweden) at embryonic day 18. The cells were tryptinized with 0.25% trypsin (Gibco, Invitrogen) for 15min at 37C, followed by trituration with 1% DNAse. Cells were seeded onto 90mm plates precoated with poly-L-lysine (10 µg/mL, Sigma) and grown in MEM-α Medium (1X) (Gibco, Invitrogen) containing 50 U/mL penicillin and 50 µg/mL streptomycin supplemented with 10% FBS. The medium was changed twice a week, and the cells were replated after 2 weeks and cultured for another week before experiments. Immunostaining with GFAP antibodies confirmed that a majority of the cells expressed GFAP (data not shown).

### DNA Constructs

Cloning of constructs encoding mouse AQP4.M23 tagged with GFP at the NH2-terminus (pGFP-AQP4.M23) and point mutation of serine 111 to alanine (S111A) in the GFP-tagged AQP4 fusion protein were performed as described previously [16].

### Cyclic AMP Measurements

Dynamic Cyclic AMP (cAMP) production in live cells was obtained by ratiometric read-out from a novel FRET-based sensor, ^T^Epac^VV^ (kindly provided by Stichting Het Nederlands Kanker lnstituut (NKI), The Netherlands) [39]. Astrocyte cell line was seeded on 30 mm coverslips in six-well plates. Cells were transfected with ^T^Epac^VV^ plasmid the following day and cultured for another 2 days before measurements. Coverslips were placed in a POCmini-2 open chamber (PeCon GmbH). Experiments were performed in oxygenated aCSF. Images were acquired using Zeiss LSM 510 confocal microscope with a 40x water immersion objective. Donor excitation was achieved with a 405nm laser; donor emission was collected between 420 and 480nm and acceptor emission between 530 and 600nm. Donor and acceptor emissions were detected simultaneously. Dynamic cAMP changes were expressed as the fluorescence ratio between donor and acceptor signals. The ratio values within the first 30 second of recordings were normalized to 1. Cells were stimulated by changing 2.5mM [K^+^]_o_ (in mM: NaCl 125, KCl 2.5, MgCl_2_ 1, NaH_2_PO_4_ 1.25, CaCl_2_ 2, NaHCO_3_ 25, Glucose 25, pH7.3-7.4) to 10mM [K^+^]_o_ (in mM: NaCl 117.5, KCl 10, MgCl_2_ 1, NaH_2_PO_4_ 1.25, CaCl_2_ 2, NaHCO_3_ 25, Glucose 25, pH7.3-7.4) or 35mM [K^+^]_o_ (in mM: NaCl 92.5, KCl 35, MgCl_2_ 1, NaH_2_PO_4_ 1.25, CaCl_2_ 2, NaHCO_3_ 25, Glucose 25, pH7.3-7.4). IBMX (3 -isobutyl-1-methylxanthine, 100 µM) was added in the aCSF to stabilize cAMP levels.

In addition, cAMP was measured using Cyclic AMP (direct) EIA Kit (Assay Design). Astrocyte primary cultures were incubated for 20min with oxygenated 2.5mM [K^+^]_o_ aCSF before experiments. The medium was changed to oxygenated aCSF containing 2.5mM [K^+^], 10mM [K^+^] or 35mM [K^+^], respectively, in the presence of 200µM IBMX. After 5min incubation, the aCSF was changed to cold 0.1M HCl, incubated for 20min and cells were scraped off. The suspensions were collected and cell lysates were centrifuged for 15min at room temperature. The measurements and analysis were performed according to the manufacturer’s instructions. Protein content in the supernatants was determined using a RC DC Protein Assay (Bio-Rad Laboratories). Each sample was analyzed as the mean value of duplicates and repeated more than 5 times.

### Water Permeability Measurements

Water permeability measurements were performed on the transfected astrocyte cell line and on primary astrocytes. The assay was performed as previously described [40]. Briefly, astrocytes grown on 40mm glass coverslips were mounted in a perfusion chamber (Focht Live Cell Chamber System) on an inverted laser scanning microscope (Zeiss LSM410). Cells were incubated with calcein AM (Molecular Probes) for 5min, then equilibrated with isoosmotic control aCSF for another 5min and exposed to an aCSF perfusion containing 2.5mM [K^+^], 10mM [K^+^] or 35mM [K^+^], respectively. The solutions were continuously bubbled with 95% O_2_/5% CO_2_ at 37^o^C. The calcein fluorescence signal was recorded immediately prior to the perfusion was switched from isoosmotic to hypoosmotic aCSF solution (in mM: NaCl 75, KCl 2.5 or NaCl 67.5, KCl 10 or NaCl 42.5, KCl 35 and MgCl_2_ 1, NaH_2_PO_4_ 1.25, CaCl_2_ 2, NaHCO_3_ 25, Glucose 25, pH7.3-7.4, respectively). Recorded series of images were analyzed off-line to obtain the time course of the calcein fluorescence in individual cells. The obtained curves, recorded during the first 7-9 seconds after exposure to the hypoosmotic solution (Figure 1A), were fitted with a single exponential function to estimate plasma membrane water permeability. In this initial phase, the rate of cell swelling is proportional to the permeability of the cellular membrane to water. The time constant of the initial region of the fluorescence curve was used as a measure of plasma membrane water permeability.

Cell swelling induced by isosmotic high potassium was evaluated in control experiments by quantification of changes in calcein fluorescence intensity at different time points relevant to the water permeability experiments (Figure 1B). Fluorescence intensity decreased in a time and concentration dependent manner with a maximum of 8.9%, indicating cell swelling. As the value of water permeability is relative, it does not depend on the absolute values of fluorescence intensity at the starting point of measurements.

We then evaluated the effect of potassium-induced cell swelling on our measurements. The fluorescence intensity curve for isosmotic high [K^+^]_o_ within the time window of water permeability recordings was isolated and the slope of intensity decrease was analyzed (Figure 1B). The slope used for off-line correction (initial 7s) and the slope for the whole recording time period (∼20s) were calculated. The differences between the slopes used for measurement correction and the slopes for the whole experimental time window were minimal, within the range of 0.00012 -0.00026, i.e. below the error value of permeability values. Thus the slope of potassium induced cell swelling during water permeability measurements was linear. This base-line slope will be adjusted for by the off-line calculations [41]. In conclusion, the evaluation of plasma membrane water permeability is not hampered by cell volume changes induced by isosmotic high potassium.

For primary cultured astrocytes, expressing endogenous AQP4, the time constant was used directly as a measure of plasma membrane water permeability. For the astrocyte cell line transfected with AQP4, the specific AQP4 water permeability was calculated as the difference between whole cell water permeability in AQP4-expressing cells and whole cell water permeability in AQP4-negative cells.

### In Vitro Phosphorylation

*In vitro* phosphorylation was performed as described previously [16]. Briefly, purified glutathione S-transferase (GST) fusion proteins, GST-APQ4 S111 or GST-APQ4 A111, were incubated with cAMP-dependent Protein Kinase (PKA), catalytic subunit (New England Biolabs) in PKA reaction buffer (New England Biolabs) containing 200 µM ATP, γ-^32^pATP (0.2 µCi/lL), and Phosphatase Inhibitor Cocktail 2 (Sigma). The reactions (30min at 30^o^C) were stopped by adding SDS-PAGE sample buffer followed by boiling for 5min. Phosphorylated proteins were isolated by SDS-PAGE Electrophoresis and visualized by autoradiography.

### Calcium Measurements

To assess free intracellular calcium ([Ca2^+^]_i_) cells were loaded with the Ca2^+^- sensitive fluorescent dye Fura-2 AM (3µM, Molecular Probes, Invitrogen) for 20min in serum-free medium at 37^o^C. Ratiometric imaging was performed using a heated chamber (FCS2, Bioptechs, Butler, PA, USA) mounted on a Zeiss Axioskop 2 microscope with a 40X/1.3 NA epifluorescent oil-immersion objective. Emission fluorescence was detected with a CCD camera (Hamamatsu ORCA-ER C4742–95) via an image-intensifier unit (Hamamatsu C9016). Fura-2 AM loaded cells were excited at wavelength 340 and 380nm and emission fluorescence was recorded with a BP510–540nm filter. All experiments were performed at 37°C using perfusion aCSF with continuous oxygenation (95% O_2_/5% CO_2_). All devices were controlled and data were analyzed using Meta-Fluor software (Molecular Devices, Downingtown, PA). For data quantifications, the calcium responses to potassium were normalized to the response to 50µM ATP.

### Drugs Used in Water Permeability- and Intracellular Calcium Measurements

Cypermethrin (5nM, 15min) from Sigma and KT 5720 (1µM, 5min) from Merck, were applied before exposure to high extracellular potassium. For [Ca2^+^]_i_ and water permeability measurements, 100µM BaCl_2_ (Sigma) was applied as indicated in the figures and was present throughout recordings. ATP was purchased from Sigma.

### Statistical Analysis

Statistical analysis was performed using Student’s t-test or, when appropriate, Mann-Whitney u-test. A difference was considered statistically significant when p<0.05.
